# Sweep imaging with Fourier transform as a tool with MRI for evaluating the effect of teriparatide on cortical bone formation in an ovariectomized rat model

**DOI:** 10.1186/s12891-021-04970-7

**Published:** 2022-01-03

**Authors:** Yasutaka Sotozono, Kazuya Ikoma, Masamitsu Kido, Okihiro Onishi, Masataka Minami, Hiroaki Wada, Shunji Yamada, Ken-Ichi Matsuda, Masaki Tanaka, Kenji Takahashi

**Affiliations:** 1 Department of Orthopaedics, Graduate School of Medical Science, Kyoto Prefectural University of Medicine, 465 Kajii-cho, Kawaramachi-Hirokoji, Kamigyo-ku, Kyoto, Japan; 2 Department of Anatomy and Neurobiology, Graduate School of Medical Science, Kyoto Prefectural University of Medicine, Kyoto, Japan

**Keywords:** Osteoporosis, Teriparatide, Sweep imaging with Fourier transform

## Abstract

**Background:**

Teriparatide (TPTD) is a drug for osteoporosis that promotes bone formation and improves bone quality. However, the effects of TPTD on cortical bone are not well understood. Sweep imaging with Fourier transform (SWIFT) has been reported as a useful tool for evaluating bound water of cortical bone, but it has yet to be used to investigate the effects of TPTD on cortical bone. This study aimed to evaluate the consequences of the effect of TPTD on cortical bone formation using SWIFT.

**Methods:**

Twelve-week-old female Sprague-Dawley rats (*n* = 36) were reared after ovariectomy to create a postmenopausal osteoporosis model. They were divided into two groups: the TPTD and non-TPTD groups. Rats were euthanized at 4, 12, and 24 weeks after initiating TPTD treatment. Tibial bones were evaluated using magnetic resonance imaging (MRI) and bone histomorphometry. In MRI, proton density-weighted imaging (PDWI) and SWIFT imaging were performed. The signal-to-noise ratio (SNR) was calculated for each method. The same area evaluated by MRI was then used to calculate the bone formation rate by bone histomorphometry. Measurements were compared using the Mann-Whitney U-test, and a *P*-value of < 0.05 was considered significant.

**Results:**

PDWI-SNR was not significantly different between the two groups at any time point (*P* = 0.589, 0.394, and 0.394 at 4, 12, and 24 weeks, respectively). Contrarily, SWIFT-SNR was significantly higher in the TPTD group than in the non-TPTD group at 4 weeks after initiating treatment, but it was not significantly different at 12 and 24 weeks (*P* = 0.009, 0.937, and 0.818 at 4, 12, and 24 weeks, respectively). The bone formation rate assessed by histomorphometry was significantly higher in the TPTD group than in the non-TPTD group at all timepoints (*P* < 0.05, all weeks). In particular, at 4 weeks, the bone formation rate was markedly higher in the TPTD group than in the non-TPTD group (*P* = 0.028, 1.98 ± 0.33 vs. 0.09 ± 0.05 μm^3^/μm^2^/day).

**Conclusions:**

SWIFT could detect increased signals of bound water, reflecting the effect of TPTD on the cortical bone. The signal detected by SWIFT reflects a marked increase in the cortical bone formation rate.

## Background

Osteoporosis is defined as “a skeletal disorder characterized by compromised bone strength predisposing to an increased risk of fracture” [1]. Bone strength is defined by bone mineral density (BMD) and bone quality, both of which are closely related to bone turnover [1, 2]. Postmenopausal osteoporosis is the most common type of osteoporosis [3], and an increase of bone turnover, bone resorption exceeding bone formation, is associated with decreased BMD and bone quality, as well as an increased risk of fracture [4]. Therefore, it is necessary to improve bone turnover to treat postmenopausal osteoporosis, and to this end, various therapeutic drugs have been developed. Teriparatide (TPTD) is a drug for the treatment of osteoporosis that promotes bone formation with intermittent administration [5]. Various studies have been conducted on the promotion of osteogenesis by TPTD [5–7]. However, the site and time at which osteogenesis occurs in the bone remain unclear.

The bone structure is composed of cortical and cancellous bones. Of the two types, the cortical bone comprises the majority, helps support body weight, and prevents fractures [8, 9]. These findings emphasize the significance of evaluating the cortical bone. However, dual-energy X-ray absorptiometry (DXA), which is commonly used for the diagnosis of osteoporosis, cannot be restricted to the cortical bone only.

Sweep imaging with Fourier transform (SWIFT) is a novel imaging method in magnetic resonance imaging (MRI) that can depict proton signals of bound water with very short T_2_ relaxation times that cannot be measured by conventional MRI [10]. It has been reported that the signals of bound water detected using SWIFT was associated with bone formation in the cortical bone of postmenopausal osteoporotic and diabetic osteoporotic rats [11, 12]. The signal-to-noise ratio (SNR) performance is better with SWIFT than with conventional imaging, although T_2_ sensitivity is the same [13]. Therefore, it is reasonable to expect that the effect of TPTD on bone formation in the cortical bone could be evaluated using SWIFT in a site-specific and time-specific manner.

This study aimed to evaluate the consequences of the effect of TPTD on cortical bone formation using SWIFT. Furthermore, we verified whether SWIFT could be used to determine the therapeutic effect of TPTD in osteoporosis.

## Methods

### Animal model

**Fig. 1:**
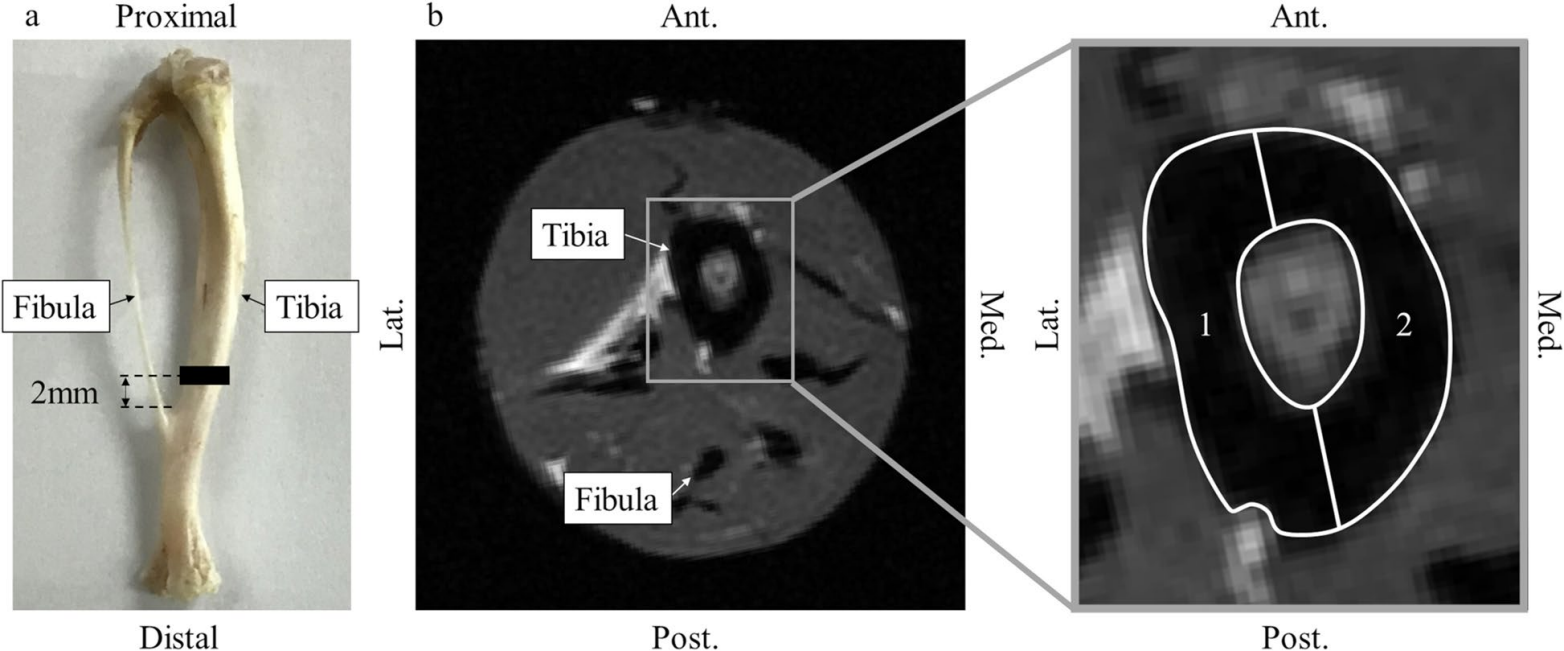
Regions of interest for MRI. **a** The regions are set 2 mm proximal to the tibiofibular union. **b** Two regions, including all the areas of cortical bone, are set in the cortical bone of the tibia in the axial plane (proton density-weighted image). Ant. = anterior, Post. = posterior, Med. = medial, Lat. = lateral

Twelve-week-old female Sprague-Dawley rats (*n* = 36; Shimizu Laboratory Supplies, Kyoto, Japan) were used in this study. The rats were kept at the animal facilities of our institute according to the Guide for the Care and Use of Laboratory Animals published by the National Institutes of Health. This study was approved by the Institutional Review Board for animal experiments and carried out in compliance with ARRIVE guidelines. Four rats were housed per transparent plastic cage. The temperature was maintained at 23–24 °C at our institution, and light-dark cycles were performed every 12 h. All animals were able to consume food and water ad libitum. All rats underwent ovariectomy under aseptic conditions, and 1.5% isoflurane was used for anesthesia. They were reared for 12 weeks after ovariectomy to create a postmenopausal osteoporosis model [14]. The rats were then randomly divided into the following two groups: TPTD and non-TPTD. For the TPTD group, TPTD was administered at 30 μg/kg/day, three times per week starting 12 weeks after the ovariectomy [6]. The non-TPTD group received normal saline at the same volume. The rats were euthanized by the intravenous injection of 4.0 mL of pentobarbital sodium (Somnopentyl®, Kyoritsuseiyaku Corp., Tokyo, Japan) at 4, 12, and 24 weeks following the initiation of TPTD treatment. Various examinations were performed according to the protocol described below. Figure 1 shows the regions of interest (ROIs) in the various tests.

### MRI

The tibia on the right side of the euthanized rats was removed and evaluated using MRI. We used a high-field MRI machine (Varian MRI system 7.04 T, Agilent Technologies, Palo Alto, CA) and a transmit-and-receive surface coil (3 × 3 mm in diameter) for animal experimentation purposes. We confirmed that the measurement errors of the MRI machine were less than 1%. A container with a sample placed at the center of the surface coil was installed. The specimens were all placed with the same orientation to ensure an identical flip angle (FA). SWIFT was performed using the following protocol: field of view (FOV) of 40 × 240 × 40 mm^3^; matrix of 256 × 256 × 256; 16 spirals; 8192 views; resolution of 0.156 × 0.938 × 0.156 mm^3^; bandwidth of 62.5 kHz; repetition time (TR) of 12.5 ms; FA of 25°; acquisition time of 4.096 ms; and scan time of 27 min 25 s. The proton density-weighted image (PDWI) was obtained according to the following protocol using a conventional fast spin echo method: FOV of 40 × 40 mm^2^; matrix of 256 × 256; resolution of 0.156 × 0.156 mm^2^; axial slice of 30; slice thickness of 1 mm; bandwidth of 100 kHz; TR of 2000 ms; echo spacing of 9.54 ms; echo train length of 4; acquisition time of 2.56 ms; and scan time of 2 min 13 s. These protocols were similar to those described in previous reports [10, 11]. The images were analyzed using a software program (ImageJ v. 1.49, National Institutes of Health, Bethesda, MD). The ROI was the entire cortical bone region of the tibia in a transverse section, which was 200 μm in thickness and 2 mm proximal to the tibiofibular union. The quality of signals in the SWIFT and PDWI were measured and averaged. The SNR was defined as the mean value of the quality of signals in the ROI/standard deviation of the quality of signals in the background, SWIFT-SNR, and PDWI-SNR, respectively.

### DXA

The left tibia was removed from euthanized rats and evaluated using DXA (DCS-600EX-R; Aloka, Tokyo, Japan). We confirmed that the measurement errors of the DXA were less than 2%. The ROI was the entire tibial region in an area, 200 μm in thickness, and 2 mm proximal to the tibiofibular union. BMD in the ROI was measured.

### Microcomputed tomography (μCT)

DXA was followed by μCT (micro-focus 2D/3D, ScanXmate-E090S40, Comscantecno, Kanagawa, Japan) of the tibia on the left side with the following settings: voltage of 60 kV, electric current of 85 μA, and voxel size of 37.5 μm. We confirmed that the measurement errors of the μCT were less than 1%. Three-dimensional reconstructed images were created using image reconstruction software (ConeCTexpress v. 1.3, Comscantecno, Kanagawa, Japan). The ROI was the entire cortical bone region of the tibia, which was the same site used for the MRI. The volumetric BMD (vBMD) of the cortical bone and the cortical bone width were measured using image analysis software (TRI/3D-BON, Ratoc System Engineering, Tokyo, Japan).

### Bone histomorphometry

Among the tibias on the right side that underwent MRI, four from each group also underwent bone histomorphometry each week. Approximately 5 mg of calcein (Sigma-Aldrich) was injected subcutaneously into the back of the tibia at 4 and 10 days prior to removal to indicate areas of the new bone. The dosing interval for calcein was 144 h (6 days). The tibia was fixed with 70% alcohol immediately after the MRI. Specimens were stained with Villanueva bone stain to assess the sites identical to those measured by MRI. The bone surface (BS, μm), porosity area (Po Ar), and cortical bone area (Ct Ar) were measured using Parfitt’s method [15]. The Haversian canal and Volkmann’s canal were included in the bone pores, and the osteocyte gap was excluded from the bone pore. Po Ar/Ct Ar (%) was measured in the cortical bone areas. BS and Ct Ar were measured under natural light at × 100 magnification and Po Ar was measured at × 200 magnification.

Under Fluorinert fluorescence, the single-labeled surface (sLs, μm), double-labeled surface (dLs, μm), and mineral apposition rate (MAR, μm/day) were measured using calcein labeling as an index. Both sLs and dLs were measured in the endosteum and periosteum. MAR was measured as the width of labeling produced by the injection at 144-h intervals in 10 selected sites at the most obvious sites of bone formation. The bone formation rate (BFR/BS, μm^3^/μm^2^/day) was measured as BFR/BS = (sLs + 2dLs) × MAR/BS. Measurements under Fluorinert fluorescence were performed at a magnification of × 200.

### Statistical analysis

Results are presented as the mean ± standard deviation. Results of DXA, μCT, MRI, and bone histomorphometry measured at each week in the TPTD and non-TPTD groups were compared using the Mann-Whitney U-test. Differences were considered significant at a *P*-value of < 0.05. EZR (Saitama Medical Center, Jichi Medical University, Saitama, Japan), a statistical software program that extends the functions of R and R Commanders [16], was used for all statistical analyses.

## Results

### MRI

**Fig. 2:**
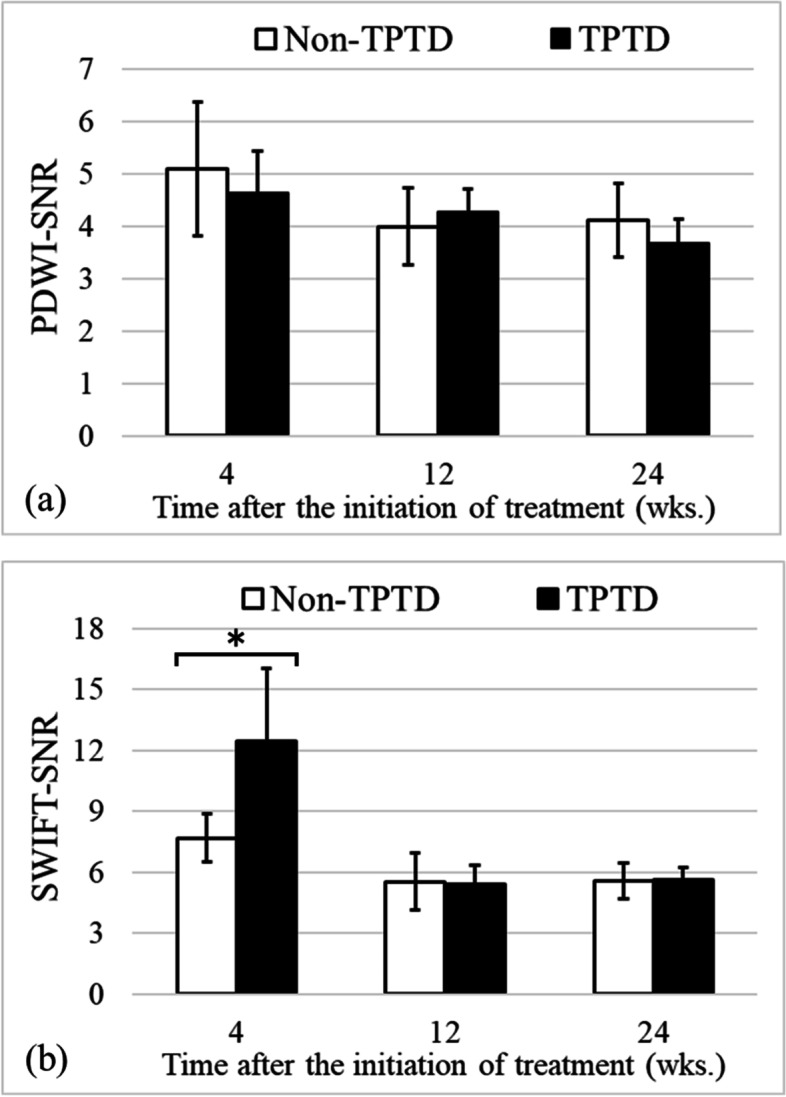
The signal-to-noise ratio (SNR) within the tibial cortical bone measured by (**a**) proton density-weighted imaging (PDWI) and (**b**) sweep imaging with Fourier transform (SWIFT) for the teriparatide (TPTD) and non-TPTD groups. Values are expressed as means ± standard deviations. There was no significant difference in the PDWI-SNR between the two groups at any time point (*P* = 0.589, 0.394, and 0.394 at 4, 12, and 24 weeks, respectively). The SWIFT-SNR was significantly higher in the TPTD group than in the non-TPTD group at 4 weeks after the initiation of treatment, but no significant difference was observed at 12 and 24 weeks (*P* = 0.009, 0.937, and 0.818 at 4, 12, and 24 weeks, respectively). *: *P* < 0.05

**Fig. 3:**
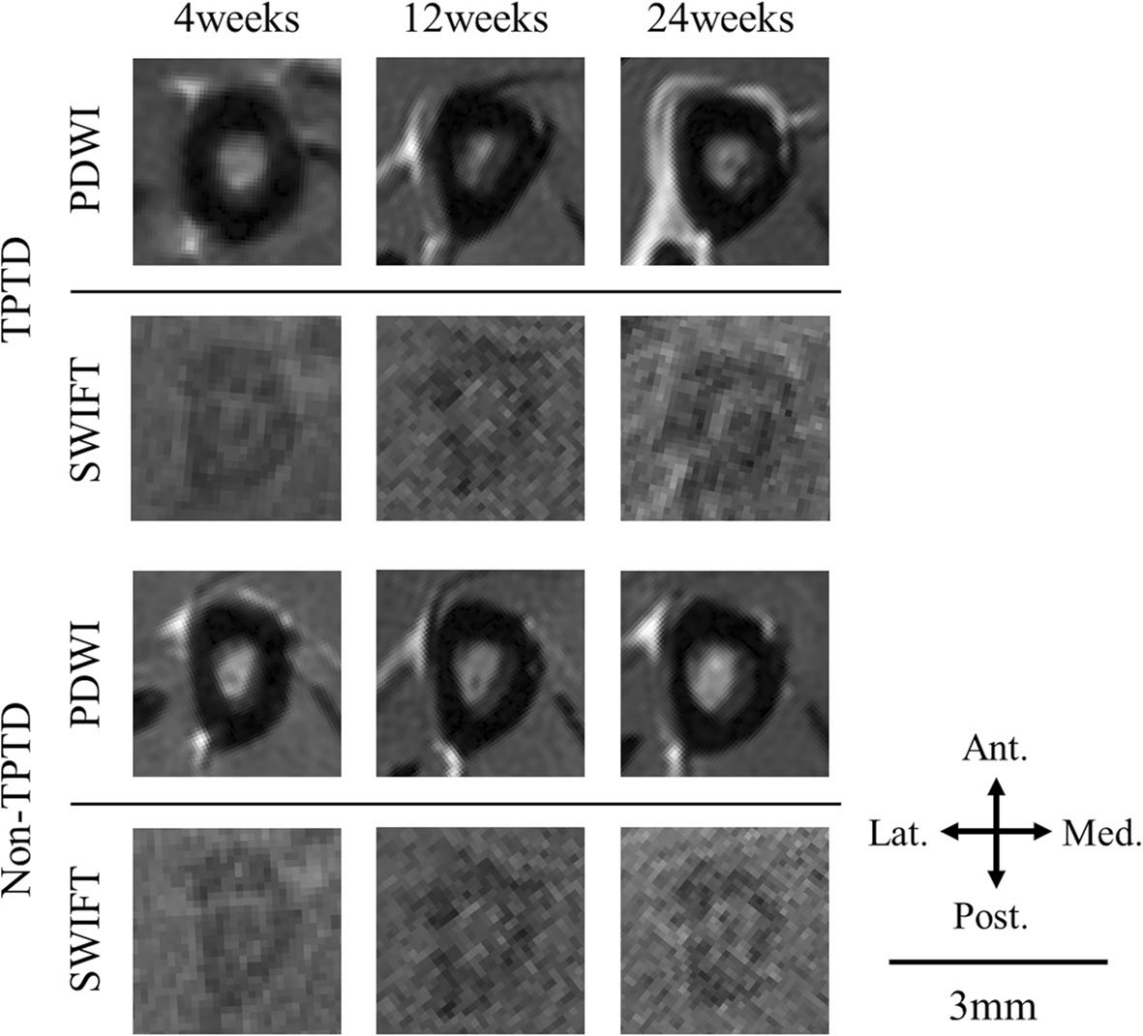
Representative images obtained via proton density-weighted imaging (PDWI) and sweep imaging with Fourier transform (SWIFT) for the teriparatide (TPTD) and non-TPTD groups. SWIFT can detect proton signals in the cortical bone, which PDWI is unable to detect. Ant. = anterior, Post. = posterior, Med. = medial, Lat. = lateral

Figure 2 shows the SNR within the cortical bone measured by MRI. The PDWI-SNRs were 4.6 ± 0.8, 4.3 ± 0.4, and 3.6 ± 0.5 in the TPTD group, and 5.1 ± 1.3, 4.0 ± 0.7, and 4.1 ± 0.7 in the non-TPTD group at 4, 12, and 24 weeks, respectively, after the initiation of TPTD treatment. There was no significant difference between the TPTD and non-TPTD groups at any time point (*P* = 0.589, 0.394, 0.394 at 4, 12, and 24 weeks, respectively). On the other hand, the SWIFT-SNRs were 12.6 ± 3.5, 5.4 ± 0.9, and 5.6 ± 0.6 in the TPTD group and 7.6 ± 1.6, 5.5 ± 1.4, and 5.6 ± 0.9 in the non-TPTD group at 4, 12, and 24 weeks, respectively. A significant difference was observed at 4 weeks after the initiation of TPTD treatment, but no significant difference was observed at 12 or 24 weeks (*P* = 0.009, 0.937, and 0.818 at 4, 12, and 24 weeks, respectively). Figure 3 shows the results of PDWI and SWIFT representatives for both groups at 4, 12, and 24 weeks after the initiation of TPTD treatment.

### DXA

The BMD in all tibial regions measured by DXA at 4, 12, and 24 weeks was 99.9 ± 4.0, 104.4 ± 5.0, and 95.3 ± 5.2 mg/cm^2^, respectively, in the TPTD group, and 89.7 ± 4.3, 98.5 ± 3.0, and 85.3 ± 2.1 mg/cm^2^, respectively, in the non-TPTD group. The levels were significantly higher in the TPTD group than in the non-TPTD group at all timepoints (*P* < 0.05 at all weeks).

### μCT

**Fig. 4:**
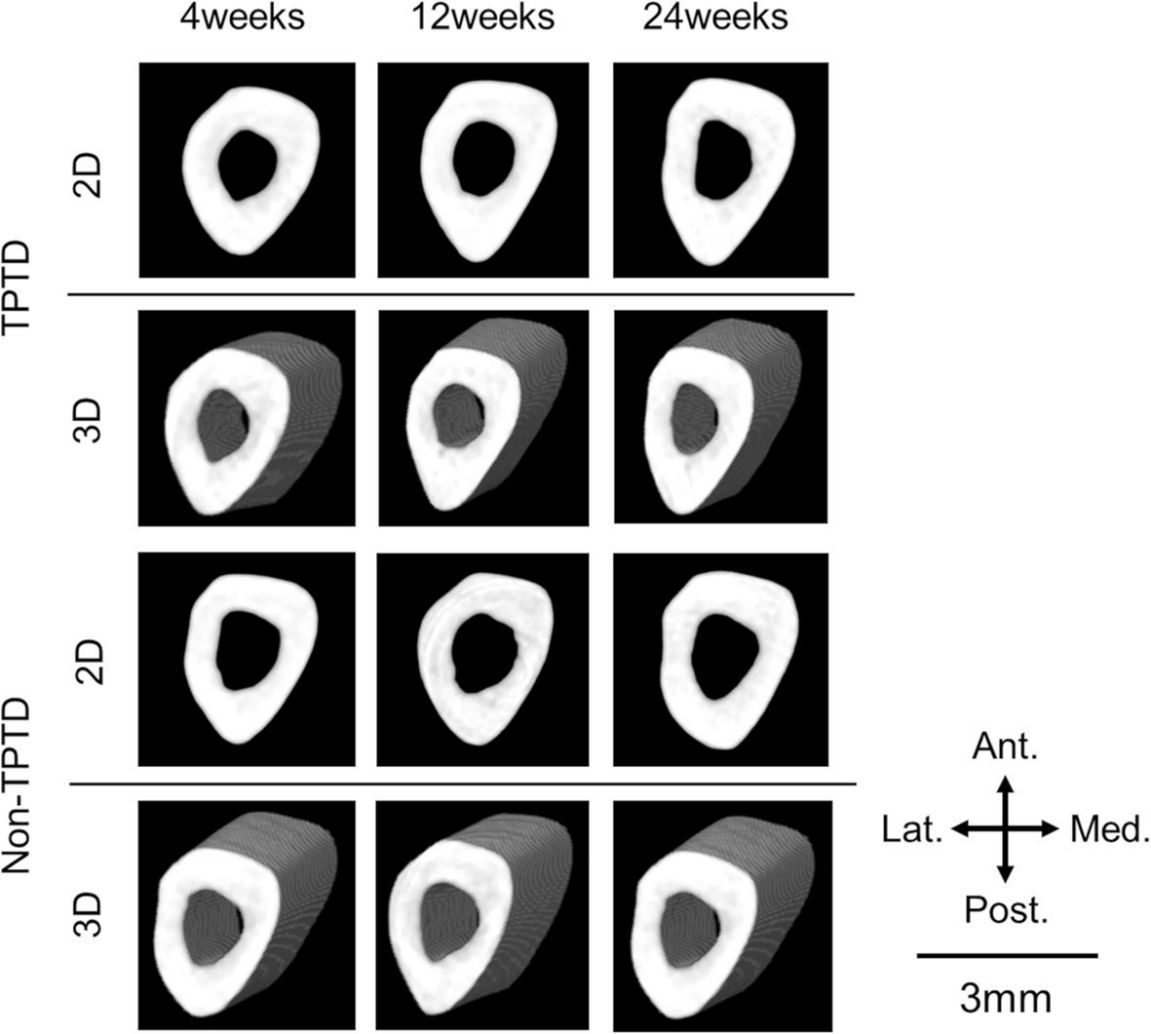
Representative 2D and 3D images obtained via microcomputed tomography (μCT) for the teriparatide (TPTD) and non-TPTD groups. Ant. = anterior, Post. = posterior, Med. = medial, Lat. = lateral

Figure 4 shows representative images obtained by μCT. The vBMDs of the cortical bone at 4, 12, and 24 weeks after the initiation of TPTD treatment were 1229 ± 71, 1231 ± 93, and 1236 ± 84 mg/cm^3^ in the TPTD group, respectively, and 1190 ± 112, 1237 ± 49, and 1223 ± 67 mg/cm^3^ in the non-TPTD group, respectively. There was no significant difference between the TPTD and non-TPTD groups at any time point (*P* = 0.699, 0.937, 0.818 at 4, 12, and 24 weeks, respectively). The cortical bone width was 760 ± 15, 786 ± 15, and 802 ± 26 μm in the TPTD group and 709 ± 16, 723 ± 13, and 715 ± 21 μm in the non-TPTD group at 4, 12, and 24 weeks, respectively, after the initiation of TPTD treatment. The levels were significantly higher in the TPTD group than in the non-TPTD group at all timepoints *(P* < 0.05 at all weeks). Table 1 shows the results of BMD measured by DXA, vBMD of cortical bone, and cortical bone width measured by μCT.

**Table 1:** Results of BMD measured by DXA, vBMD of cortical bone, and cortical width measured by μCT

	Time after the initiation of treatment	Non-TPTD	TPTD	*P*-value
DXA				
BMD (mg/cm^2^)	4 weeks	89.7 ± 4.3	99.9 ± 4.0	< 0.01
	12 weeks	98.1 ± 3.0	104.4 ± 5.0	< 0.01
	24 weeks	85.3 ± 2.1	95.3 ± 5.2	< 0.05
μCT				
vBMD of cortical bone (mg/cm^3^)	4 weeks	1190 ± 112	1229 ± 71	0.67
	12 weeks	1237 ± 49	1231 ± 93	0.94
	24 weeks	1223 ± 67	1235 ± 84	0.82
Cortical width (μm)	4 weeks	709 ± 16	760 ± 15	< 0.01
	12 weeks	723 ± 13	786 ± 15	< 0.01
	24 weeks	714 ± 22	801 ± 26	< 0.01

Values are expressed as means ± standard deviations. *BMD* Bone mineral density, *DXA* Dual-energy X-ray absorptiometry, *vBMD* Volumetric bone mineral density, *μCT* Microcomputed tomography, *TPTD* Teriparatide

### Bone histomorphometry

**Fig. 5:**
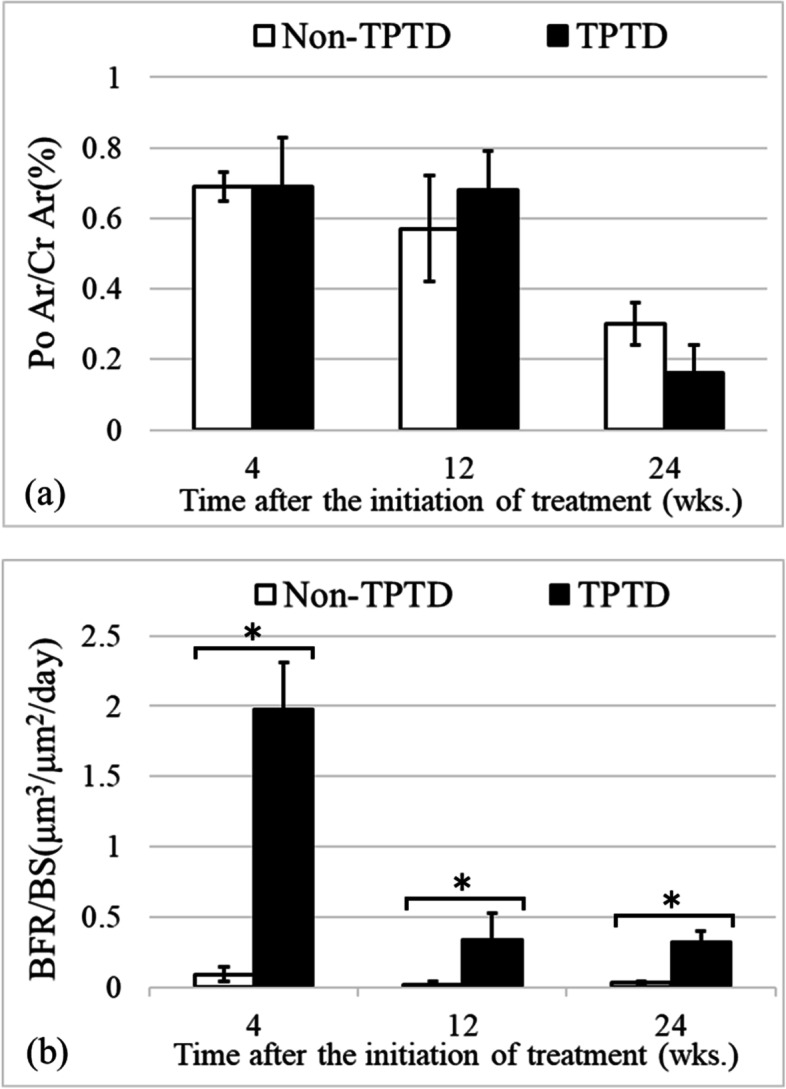
**a** Porosity area/cortical area (Po Ar/Ct Ar) and **b** bone formation rate (BFR/BS) in the tibial cortical bone measured by bone histomorphometry. There was no significant difference in Po Ar/Ct Ar between the teriparatide (TPTD) and non-TPTD groups at any time point (*P* = 0.857, 0.486, and 0.0571 at 4, 12, and 24 weeks, respectively). BFR/BS was significantly higher in the TPTD group than in the non-TPTD group at all timepoints (*P* < 0.05 at all weeks). In particular, at 4 weeks, BFR/BS was markedly higher in the TPTD group than in the non-TPTD group. *: *P* < 0.05

**Fig. 6:**
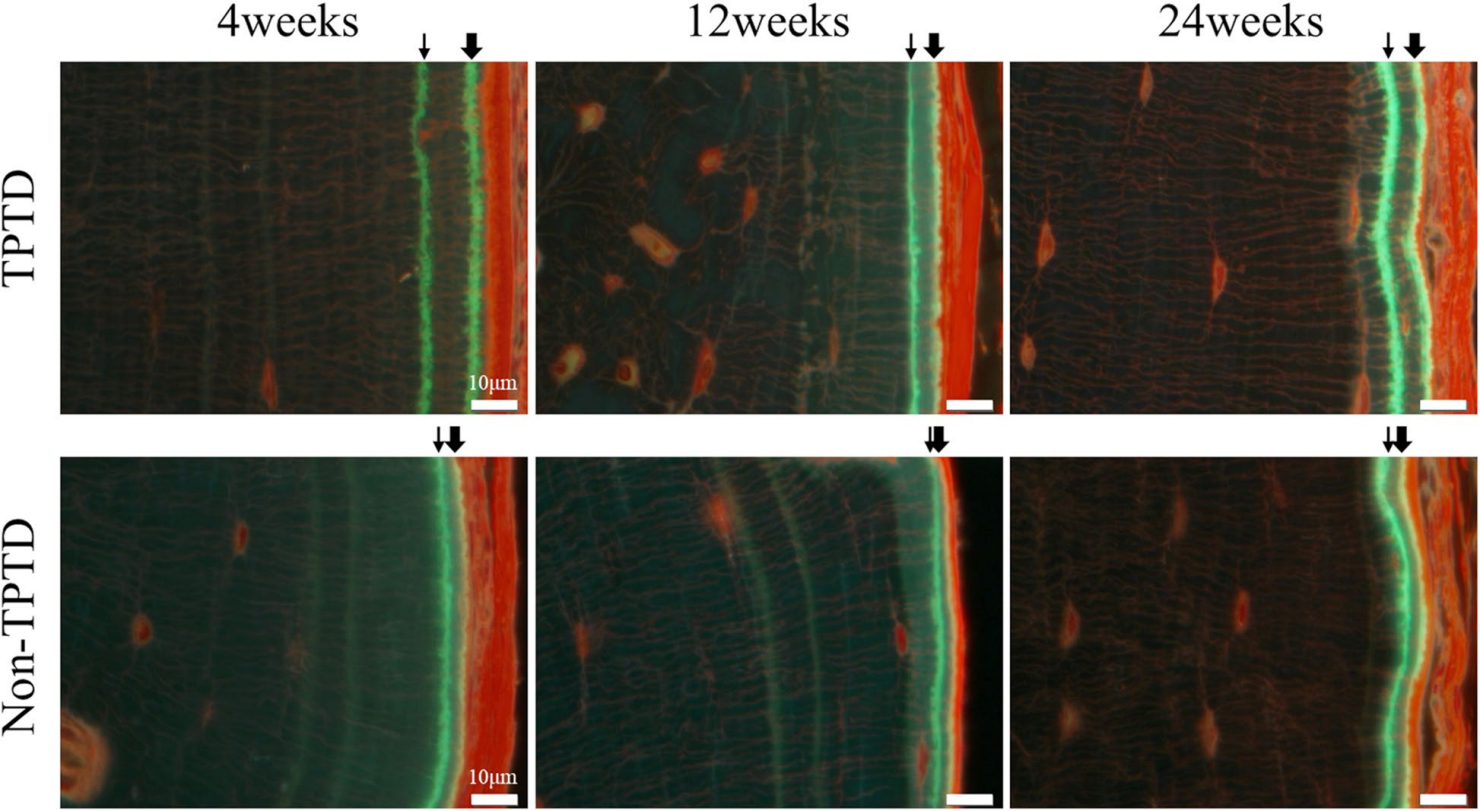
Representative images of the areas of the new bone in the endosteum when observed under fluorescent light. The thin arrow indicates calcein injected at 10 days before tibial removal, and the thick arrow indicates calcein injected at 4 days before tibial removal. A marked increase in the bone formation rate in the teriparatide (TPTD) group was observed at 4 weeks after the initiation of TPTD treatment

Figure 5 shows Po Ar/Ct Ar and BFR/BS measured by bone histomorphometry. Po Ar/Ct Ar was 0.69 ± 0.14, 0.68 ± 0.11, and 0.16 ± 0.08 in the TPTD group, and 0.69 ± 0.04, 0.57 ± 0.15, and 0.30 ± 0.06 in the non-TPTD group at 4, 12, and 24 weeks, respectively, after the initiation of TPTD treatment. There was no significant difference between the TPTD and non-TPTD groups at any time point (*P* = 0.857, 0.486, and 0.057 at 4, 12, and 24 weeks, respectively). BFR/BS was 1.98 ± 0.33, 0.34 ± 0.19, and 0.32 ± 0.08 μm^3^/μm^2^/day in the TPTD group, and 0.09 ± 0.06, 0.02 ± 0.02, and 0.03 ± 0.01 μm^3^/μm^2^/day in the non-TPTD group at 4, 12, and 24 weeks, respectively, after the initiation of TPTD treatment. The values were significantly higher in the TPTD group than in the non-TPTD group at all timepoints (*P* < 0.05 at all weeks). In particular, at 4 weeks, BFR/BS was markedly higher in the TPTD group than in the non-TPTD group. Figure 6 shows representative images of the bone formation rate.

## Discussion

Our results suggest that it was possible to detect signals representing the effect of TPTD on cortical bone formation using SWIFT. The SWIFT-SNR was markedly increased early after the initiation of TPTD treatment, similar to the change in the bone formation rate in bone histomorphometry. We believe that TPTD promotes bone formation in the cortical bone to a greater extent in the early stage after the initiation of TPTD treatment, and SWIFT detects the signals emitted by the newly formed bone area. This change could not be detected using PDWI, a conventional MRI technique. The effects of TPTD on the cortical bone could be detected using DXA and μCT; however, a marked increase of bone formation early after the initiation of TPTD treatment could not be determined. The use of SWIFT enables the evaluation of the effect of TPTD on signals of bound water in a site- and time-specific manner.

DXA, which measures BMD, is the most commonly performed test for the treatment of osteoporosis [17]. The measurement results in this study were higher in the TPTD group than in the non-TPTD group, as in previous reports [18], confirming the appropriate administration of TPTD. DXA is a two-dimensional assessment method, and the measured BMD combines the cortical and cancellous bones. Therefore, it is not possible to only assess the cortical bone with DXA, even though the cortical bone has an important role in bone strength.

μCT is a three-dimensional assessment method that can be used to evaluate the cortical and cancellous bones separately [19]. Although the results of this study showed that the vBMD of the cortical bone did not differ at all weeks, the cortical bone width was higher in the TPTD group at all weeks, similar to previous reports [20]. TPTD may accelerate the cortical bone formation and increase the cortical bone width. μCT can be used to focus on the cortical bone. However, μCT is a mineral-based histomorphological assessment method and is therefore unable to assess non-mineral items.

Similar to μCT, MRI is another three-dimensional assessment method. However, while μCT is used to assess minerals, MRI may be used to assess proton signals. The proton signal obtained from the cortical bone was from free and bound water [21]. The proton signal from free water has a millisecond T_2_ relaxation time and can be detected using PDWI, a conventional MRI method. On the other hand, the proton signal from bound water had a T_2_ relaxation time of microseconds and could not be detected by PDWI [22]. In contrast, SWIFT can be used to detect signals with very short T_2_ relaxation times, allowing the detection of signals of bound water [10]. In this study, although the PDWI-SNR showed no differences at all weeks, the SWIFT-SNR was higher in the TPTD group than in the non-TPTD group only at 4 weeks after the initiation of TPTD treatment. It was possible to detect the effect of TPTD as a signal of an increase in the amount of bound water in the cortical bone early after the initiation of TPTD treatment.

TPTD has been reported to greatly improve bone formation in the early phase after treatment initiation [7, 23]. With SWIFT, high signals were also detected at 4 weeks after the initiation of TPTD treatment, and the formation rate of the cortical bone in bone histomorphometry was also high at 4 weeks after the initiation of TPTD treatment compared with other weeks. We believe that the signal detected by SWIFT reflects a marked increase in the bone formation rate in the cortical bone. Considering that bound water is abundant in the collagen in new bone areas and decreases with mineralization [24], the increase in the bone formation rate should be captured in SWIFT by detecting the signal of the bound water that exists in the collagen of new bone areas. From the present results, SWIFT seems to be more suitable to detect initial cortical bone formation than other imaging methods, such as PDWI, CT, and DXA. However, the increase in bone formation rate in the TPTD group at 12 and 24 weeks after the initiation of TPTD treatment could not be evaluated by SWIFT, possibly because the slight increase in the bone formation rate may have been insufficient to detect a signal difference.

A previous study has reported that the effect of TPTD on cortical bone porosity depends on the frequency of administration, increasing with daily administration and having no effect or decreasing with weekly administration [25]. In this study, TPTD was administered to rats at a frequency of one dose per week on a human equivalent dose basis [6]; the cortical bone porosity decreased, and the cortical bone width increased, as in previous reports [26]. The results of bone histomorphometry in this study correctly reflect the effects of TPTD on the cortical bone.

Other methods for assessing bone formation include bone formation markers by blood sampling and bone histomorphometry by biopsy. Bone formation markers reflect the metabolism of the whole bone; therefore, site-specific evaluation was not possible. Furthermore, it is difficult to accurately evaluate the values due to the influence of renal function [27]. Although bone histomorphometry by biopsy can accurately evaluate bone formation, it is necessary to extract the region to be evaluated, which makes it highly invasive. Furthermore, the sample obtained by extraction was also small, and the amount of information was lacking [28]. SWIFT is a non-invasive method that could detect signals reflecting bone formation in a site- and time-specific manner, and it may be used to assess bone formation as an alternative method to bone formation markers or bone histomorphometry by biopsy.

This study had two limitations. First, slight increases in the bone formation rate may not have been detectable as differences in SWIFT signals. Because the rate itself was slow, the proportion of the new bone areas in the cortical bone was small; thus, we believe that the difference in the SWIFT signal could not be detected. We recommend verifying the extent of the bone formation rate that can be detected as a signal in the future. Second, bone formation markers were not measured. Bone formation markers are commonly used to assess bone formation and should be measured to compare the results of SWIFT with those of conventional test. However, we believe that the measurement by bone histomorphometry was sufficient to evaluate the bone formation rate.

## Conclusions

We found that SWIFT could detect increased signals of bound water, reflecting the effect of TPTD on the cortical bone. The signal detected by SWIFT reflects a marked increase in the cortical bone formation rate. We believe that the use of SWIFT to assess bone formation in the cortical bone might help determine the therapeutic effect of TPTD on osteoporosis.

## Data Availability

The datasets analyzed during the current study are available from the corresponding author on reasonable request.

## Abbreviations

BMD: Bone mineral density
TPTD: Teriparatide
DXA: Dual-energy X-ray absorptiometry
SWIFT: Sweep imaging with Fourier transform
MRI: Magnetic resonance imaging
SNR: Signal-to-noise ratio
ROIs: Regions of interest
FA: Flip angle
TR: Repetition time
FOV: Field of view
PDWI: Proton density-weighted image
μCT: Microcomputed tomography
vBMD: Volumetric bone mineral density
BS: Bone surface
Po Ar: Porosity area: Ct Ar: Cortical bone area
sLs: Single-labeled surface
dLs: Double-labeled surface
MAR: Mineral apposition rate
BFR/BS: Bone formation rate
